# Health insurance coverage, type of payment for health insurance, and reasons for not being insured under the National Health Insurance Scheme in Ghana

**DOI:** 10.1186/s13561-019-0255-5

**Published:** 2019-12-29

**Authors:** Martin Amogre Ayanore, Milena Pavlova, Nuworza Kugbey, Adam Fusheini, John Tetteh, Augustine Adoliba Ayanore, James Akazili, Philip Baba Adongo, Wim Groot

**Affiliations:** 1 Department of Health Policy Planning and Management, School of Public Health, University of Health and Allied Sciences, Ho, Ghana; 2 Department of Health Services Research, CAPHRI, Maastricht University Medical Centre, Faculty of Health, Medicine and Life Sciences, Maastricht University, Maastricht, The Netherlands; 3 Department of Family and Community Health, School of Public Health, University of Health and Allied Sciences, Ho, Ghana; 4 Department of Preventive and Social Medicine, Dunedin School of Medicine, University of Otago, Dunedin, New Zealand; 5 Department of Community Health, University of Ghana Medical School, School of Public Health, College of Health Sciences, University of Ghana, Accra, Ghana; 6 Centre for Health Policy Advocacy Innovation & Research in Africa (CHPAIR-Africa), Accra, Ghana; 7 Ghana Health Service Research Division, Accra, Ghana; 8 Department of Social and Behavioral Science, School of Public Health, University of Ghana, Accra, Ghana; 9 Top Institute Evidence-Based Education Research (TIER), Maastricht University, Maastricht, The Netherlands

**Keywords:** Health insurance coverage, Type of payment, Insured and uninsured, National Health Insurance Scheme (NHIS), Ghana

## Abstract

**Background:**

Ghana’s National Health Insurance Scheme has improved access to care, although equity and sustainability issues remain. This study examined health insurance coverage, type of payment for health insurance and reasons for being uninsured under the National Health Insurance Scheme in Ghana.

**Methods:**

The 2014 Ghana Demographic Health Survey datasets with information for 9396 women and 3855 men were analyzed. The study employed cross-sectional national representative data. The frequency distribution of socio-demographics and health insurance coverage differentials among men and women is first presented. Further statistical analysis applies a two-stage probit Hackman selection model to determine socio-demographic factors associated with type of payment for insurance and reasons for not insured among men and women under the National Health insurance Scheme in Ghana. The selection equation in the Hackman selection model also shows the association between insurance status and socio-demographic factors.

**Results:**

About 66.0% of women and 52.6% of men were covered by health insurance. Wealth status determined insurance status, with poorest, poorer and middle-income groups being less likely to pay themselves for insurance. Women never in union and widowed women were less likely to be covered relative to married women although this group was more likely to pay NHIS premiums themselves. Wealth status (poorest, poorer and middle-income) was associated with non-affordability as a reason for being not insured. Geographic disparities were also found. Rural men and nulliparous women were also more likely to mention no need of insurance as a reason of being uninsured.

**Conclusion:**

Tailored policies to reduce delays in membership enrolment, improve positive perceptions and awareness of National Health Insurance Scheme in reducing catastrophic spending and addressing financial barriers for enrolment among some groups can be positive precursors to improve trust and enrolments and address broad equity concerns regarding the National Health Insurance Scheme.

## Introduction

Ghana’s National Health Insurance Scheme (NHIS) has been operational since 2003. The aim of the NHIS is to ensure access for all and equity in the use of health care services by removing financial barriers to access at the point of use [1]. In 2014, about 10.5 million Ghanaians were covered under the NHIS (an estimated 40% of the population), with inpatient and outpatient visits to health facilities rising from 0.5 per capita to about 3 per capita between 2005 and 2014 [2].

One financing mechanism of the NHIS is direct payment of premiums for membership. Direct payment of premiums is primarily used by informal sector workers [3]. Formal sector worker’s contributions for the NHIS come from social security contributions paid both by the worker and the employer. Premium payers have to pay and renew membership annually to benefit from the scheme. In addition to the direct premium payments, there is a 2.5% Health Insurance Levy on selected goods and services that is used to fund the NHIS. Under Ghana’s current NHIS policy (Act 852), pregnant women, indigents, persons with mental disorders, social security recipients and pensioners, elderly (above 70 years) and differently-abled persons as determined by the Ministry of Gender, are exempted from premium payment [4].

Enrolment into the NHIS has been reported to increase with the education level of the head of the household [5]. Socio-demographic and spatial differentials are also reported regarding subscription to the NHIS [6]. The insured also pay out-of-pocket (OOP) payments for health care [7, 8]. These OOP payments potentially influence current and future enrolment under the scheme. Poor claim returns, poor accountability and non-transparent operations have been found to be threats to enrolment [9]. Poor quality and low satisfaction ratings by clients under the NHIS are reported [10]. Despite these concerns, Ghana’s NHIS has improved access to a continuum of care [11, 12]. The obstacle in many poor resource settings is how to ensure that users who enroll to the NHIS do not fall back into conditions that will necessitate OOP payments due to ill health. Investigating individual and household familial factors that are associated with payments for health insurance is therefore important.

Spatial and household determinants of health insurance subscription are reported as well [6]. The effect of education and socio-economic status on NHIS enrolment has been examined in studies in Ghana [13–17]. Willingness to pay for insurance among informal sector workers [18] and what socio-demographic groups pay to enroll under the national health insurance have been investigated [8].

Despite previous studies on NHIS enrolment in Ghana [13–17], limited evidence exists on how persons insured under the NHIS, pay for their enrollment and why some population groups remain uninsured under the NHIS. This study explicitly focuses on the above literature gap and analyzes a national representative data to further investigate these issues. In particular, the aim of the study is to examine health insurance coverage, type of payment for health insurance and reasons for being uninsured under the NHIS in Ghana. The evidence presented in this study on type of payment for NHIS membership and reasons for being uninsured differentiates this study from previous research on the NHIS in Ghana. Findings from this study are relevant to inform why some population groups lag behind with regards to health insurance coverage. Also, the findings highlight equity concerns in health care access and implications for Ghana’s health care system as efforts to accelerate the progress towards Universal Health Coverage (UHC).

## Methods

### Data

The 2014 GDHS datasets for women and men were analyzed. Demographic Health Surveys (DHS) ensure national representation by employing a two-stage sample design across all geographical regions. A total of 427 clusters were selected for the survey which comprises 216 urban and 211 rural areas from enumeration areas (EAs) defined by the 2010 Population and Housing Census. A total of 12,831 households across the 10 regions in Ghana were selected for the 2014 survey. For the individual-level data, women in reproductive-age women (15–49 years) were included as well as men aged 15–59 years. Survey data for women aged 15–49 were collected in 11,835 occupied households, while data for men aged 15–59 were collected in half of all sampled households [19]. No reason was provided in the original data report why more women were sampled than men.

Both women and men datasets contain information on respondents’ background characteristics, HIV testing and knowledge, anthropometric measures (height/weight), anemia status, fertility preferences, child health outcomes and health insurance measures. The women dataset also contains data on reproductive history and maternal and child health outcomes not included in the male dataset [19]. In the interviewed households, a total of 9656 eligible women were identified. Interviews were however conducted among a total of 9396 women, providing a response rate of 97%. In addition, 4609 eligible men were identified while 4388 of them were interviewed, providing a response rate of 95% [19]. To ensure data comparability men and women aged 15–49 were analyzed, i.e. men aged 50–59 were excluded. Thus, the total study samples consisted of 9396 women and 3855 men.

### Dependent variables

Three outcomes were assessed: health insurance coverage, type of payment for insurance and reasons why some individuals were not registered for health insurance. The three outcomes were of interest because previous literature indicated that NHIS coverage and not being enrolling to the NHIS vary across population groups and regions [1, 20], which we investigated further in our analysis. Health insurance coverage was dichotomized as 0 = no for not covered and 1 = yes for those covered. Regarding type of payment, the question, who paid for national health insurance membership was applied. This was recoded into 2 categories based on the original dataset; 1 = paid self; 0 = paid by others (relative/friend; employer; the state/exempted). Regarding the third outcome measure, the question, why not registered with national health insurance was asked. Three dummy variables were created: cannot afford premium (0 = no, 1 = yes), do not trust the national health insurance (0 = no, 1 = yes) and do not need health insurance (0 = no, 1 = yes). Thus, we used 5 dependent variables in total.

### Independent variables

The inclusion of independent variables is based on previous literature on NHIS enrolments in Ghana [6, 13–17]. Eight socio-demographic level variables were included in the analysis: age, marital status, a wealth index, region, educational level, religion, place of residence and parity levels. Maternal age was categorized in five-year group intervals (15–19, 20–24, 25–29, 30–34, 35–39, 40–44, 45–49). Male age groups included 2 additional groups; 50–54, and 55–59 in addition to what is categorized for women. To ensure uniformity in comparisons, only respondents aged between 15 and 49 years were included. For both men and women datasets, marital status was recoded into three responses; never in union, married and widowed. An available wealth index in the dataset was used (poorest, poorer, middle, richer and richest). Region (10 geographical divisions) and educational level (no education, primary, secondary and higher) as coded in the dataset was used. Religion was recoded into responses (Christianity, Islam, Traditional and no religion), parity was recoded into responses; nulliparous, 1–2 births, 3–4 births and 5+ births. Urban and rural classification was used for place of residence.

### Statistical analysis

First, descriptive analysis for socio-demographic characteristics for both male and female and health insurance coverage was performed and results were presented in the form of frequencies and a graph. Further statistical analysis was performed on the dependent outcome variables of interest, namely type of payment for NHIS (paid by self and paid by others) and the three reasons for not being insured under the NHIS (cannot afford NHIS, yes/no, do not trust NHIS, yes/no and do not need NHIS yes/no). We used a two-stage probit Hackman selection model. We applied Heckman selection in this estimation to control for selection bias for type of payment (insured group) and reasons not insured (uninsured group). Thus, the selection equations for type of payment was NHIS status (1 = covered and 0 = not covered) and for reasons for being uninsured, NHIS status was recoded as 1 = not covered and 0 = covered. Statistical significance threshold of *p* < .05 and lower was applied in all analysis. Education status was used as instriúmental variable based on the preliminary anaylsis. Software package Stata version 14 was used to perform the analyses.

## Results

### Health insurance coverage

Table 1 presents socio-demographic and health insurance coverage differentials among men and women. A relatively high proportion of male and female respondents were in the age group 15–19 years. About a quarter of male and female respondents were within the poorest income group. A quarter of women (24.3%) and less than a quarter of men (13.0%) had no formal education. More than half of men and women were educated at the secondary level and married, while more than half of both sample groups resided in rural areas. The majority of men (83.8%) and women (74.7%) were employed. On average, 66.0% of women and 52.6% of men were covered under the NHIS.

**Table 1:** Socio-demographics of men and women included in the study

Demographic variables	Men	Women
	*N* = 3855	*N* = 9396
	N (%)	N (%)
Age in 5-year groups		
15–19	889 (23.1)	1756 (18.7)
20–24	620 (16.1)	1571 (16.7)
25–29	577 (15.0)	1564 (16.6)
30–34	497 (12.9)	1343 (14.4)
35–39	472 (12.2)	1260 (13.4)
40–44	442 (11.5)	1032 (11.0)
45–49	358 (9.3)	870 (9.3)
Marital status		
Never in union	1854 (48.1)	3041 (32.4)
Married	1910 (49.6)	5826 (62.0)
Widowed	91 (2.4)	529 (5.6)
Wealth index		
Poorest	990 (25.7)	2335 (24.9)
Poorer	717 (18.6)	1759 (18.7)
Middle	735 (19.1)	1902 (20.2)
Richer	726 (18.8)	1771 (18.9)
Richest	687 (17.8)	1629 (17.3)
Educational level		
No education	502 (13.0)	2281 (24.3)
Primary	636 (16.5)	1747 (18.6)
Secondary	2328 (60.4)	4854 (51.7)
Higher	389 (10.1)	514 (5.5)
Religion		
Christian	2604 (67.6)	7171 (76.3)
Islam	823 (21.4)	1726 (18.4)
Traditional/spiritualist	210 (5.5)	226 (2.4)
No religion	218 (5.7)	273 (2.9)
Place of residence		
Urban	1826 (47.4)	4602 (49.0)
Rural	2029 (52.6)	4794 (51.0)
Parity		
No Child	1935 (50.2)	2948 (31.4)
1–2	791 (20.5)	2747 (29.2)
3–4	643 (16.7)	2122 (22.6)
5+	486 (12.6)	1579 (16.8)
Employment status		
Unemployed	624 (16.2)	2376 (25.3)
Employed	3231 (83.8)	7020 (74.7)

**Fig. 1:**
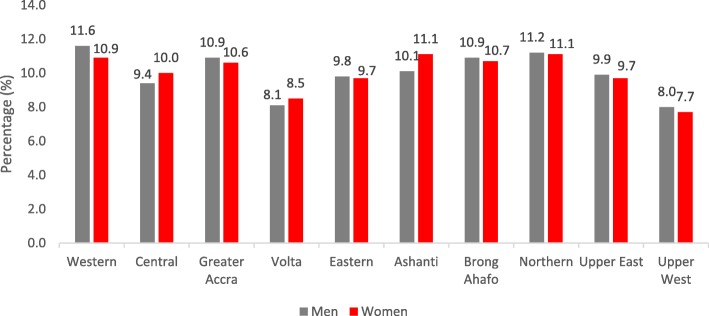
NHIS coverage per regions segregated by sex

In Table 2, we also observed geographical differences regarding NHIS coverage across regions among men and women. Specifically, in the Western region, men more frequently had insurance coverage compared to women (11.6% men and 10.9% women), while the situation was reverse in the Ashanti region (11.1% women and 10.1% men) and the Central region (10.0% women and 9.4% men) (see Fig. 1).

**Table 2:** Health insurance coverage differentials among men and women

Demographic variables	Men	Women
	*N* = 3855	*N* = 9396
	N (%)	N (%)
Covered by health insurance		
No	1827 (47.4)	3196 (34.0)
Yes	2028 (52.6)	6200 (66.0)
Why not registered with NHIS:	*N* = 1827	*N* = 3196
Cannot afford premium		
No	1325 (72.5)	2364 (74.0)
Yes	502 (27.5)	832 (26.0)
Do not trust		
No	1690 (92.5)	3089 (96.7)
Yes	137 (7.5)	107 (3.4)
Don’t need health insurance		
No	1436 (78.6)	2852 (89.2)
Yes	391 (21.4)	344 (10.8)
Pay NHIS membership	*N* = 2028	*N* = 6200
Yes, paid myself	1182 (58.3)	2281 (36.8)
Yes, paid by a relative /friend	736 (36.3)	3544 (57.2)
Yes, paid by employer/SSNIT	103 (5.1)	118 (1.9)
No, exempt as indigent	7 (0.4)	257 (4.2)

A quarter of men and women were not insured because of non-affordability of health insurance premiums (see Table 2). About 7.5% of uninsured men and 3.4% of uninsured women indicated they were uninsured because they did not trust the NHIS. About 21.4% of uninsured men and 10.8% of uninsured women mentioned they did not need health insurance. Regarding payment for NHIS membership, about half of the men who were insured, paid themselves while about half of women who were insured, got insurance through payments from a friend or relative. About 0.4% of insured men and 4.2% of insured women were exempted from making direct premium payments to be enrolled under the NHIS.

### Socio-demographic characteristics and health insurance coverage

Compared to men aged 30–34 years, men aged 15–19, 35–49 years and 45–49 years were more likely to be covered by health insurance (see Table 3). Among women, those aged 20–24 years were less likely to be covered for NHIS. Women never in union and those widowed were less likely to be covered for health insurance, compared to married women respectively. Wealth status (poorest, poorer, middle and richer) was associated with the lack of NHIS coverage (see Table 3).

**Table 3:** Probit Hackman model showing type of payment for insurance among NHIS insured men and women in Ghana

Variables	Male		Female	
	NHIS status 1 = covered, 0 = not covered (selection)	Type of payment (paid by self = 1, paid by others = 0)	NHIS status 1 = covered, 0 = not covered (selection)	Type of payment (paid by self = 1, paid by others = 0)
	β (SE)	β (SE)	β (SE)	β (SE)
Age				
15–19	0.358*** (0.105)	−1.409*** (0.154)	− 0.0989 (0.0707)	−1.593*** (0.122)
20–24	0.0472 (0.0981)	−0.624*** (0.130)	−0.204*** (0.0585)	−0.590*** (0.0700)
25–29	− 0.100 (0.0852)	−0.0585 (0.112)	− 0.0310 (0.0521)	−0.148* (0.0577)
30–34	ref	ref	ref	ref
35–39	0.285** (0.0874)	0.127 (0.111)	0.0494 (0.0537)	0.128* (0.0595)
40–44	0.184* (0.0922)	0.0387 (0.121)	0.00825 (0.0575)	0.223** (0.0692)
45–49	0.360*** (0.0997)	0.0659 (0.135)	0.0688 (0.0611)	0.424*** (0.0752)
Marital status				
Never in union	−0.191 (0.0994)	− 0.367** (0.136)	−0.234*** (0.0554)	0.265** (0.0814)
Married	ref	ref	ref	ref
Widowed	−0.270 (0.141)	− 0.352* (0.176)	−0.194** (0.0607)	0.789*** (0.131)
Wealth index				
Poorest	−0.746*** (0.109)	−0.244 (0.150)	− 0.510*** (0.0718)	−0.224* (0.107)
Poorer	−0.581*** (0.0963)	−0.268* (0.134)	−0.424*** (0.0629)	−0.131 (0.0982)
Middle	−0.509*** (0.0855)	−0.165 (0.122)	− 0.353*** (0.0548)	−0.0109 (0.0844)
Richer	−0.266*** (0.0759)	−0.0650 (0.0993)	− 0.216*** (0.0493)	0.0393 (0.0686)
Richest	ref	ref	ref	ref
Region				
Western	0.321*** (0.0948)	0.296* (0.134)	0.382*** (0.0606)	0.220* (0.0855)
Central	0.222* (0.0993)	0.279 (0.144)	0.0228 (0.0611)	0.0674 (0.0846)
Greater Accra	ref	ref	ref	ref
Volta	0.970*** (0.108)	0.863*** (0.151)	0.672*** (0.0682)	0.650*** (0.0956)
Eastern	0.607*** (0.0979)	0.677*** (0.142)	0.513*** (0.0638)	0.120 (0.101)
Ashanti	0.537*** (0.0942)	0.818*** (0.127)	−0.0315 (0.0582)	− 0.128 (0.0796)
Brong Ahafo	0.936*** (0.0989)	0.845*** (0.138)	0.841*** (0.0649)	− 0.106 (0.140)
Northern	1.120*** (0.110)	0.720*** (0.159)	0.811*** (0.0718)	0.175 (0.127)
Upper east	1.623*** (0.116)	1.290*** (0.162)	0.816*** (0.0707)	0.336** (0.124)
Upper west	1.579*** (0.117)	1.033*** (0.163)	1.439*** (0.0808)	0.418** (0.160)
Religion				
Christian	−0.134* (0.0596)	−0.154* (0.0738)	−0.152*** (0.0417)	0.160** (0.0525)
Islam	ref	ref	ref	ref
Traditional/spiritualist	−0.577*** (0.106)	−0.295 (0.158)	− 0.362*** (0.0962)	0.337* (0.140)
No religion	−0.535*** (0.106)	−0.430* (0.175)	−0.359*** (0.0860)	−0.0119 (0.128)
Residence				
Urban	ref	ref	ref	ref
Rural	0.105 (0.0582)	−0.0126 (0.0781)	0.0158 (0.0379)	−0.0107 (0.0476)
Parity				
No child	0.0390 (0.124)	−0.139 (0.168)	0.116 (0.0721)	0.253** (0.0890)
1–2	−0.0325 (0.0878)	− 0.228 (0.123)	0.214*** (0.0521)	0.392*** (0.0614)
3–4	0.0473 (0.0791)	− 0.141 (0.108)	0.134** (0.0463)	0.147** (0.0553)
5+	ref	ref	ref	ref
Employment				
Unemployed	0.391*** (0.0688)	−0.423*** (0.0961)	0.0602 (0.0364)	− 0.403*** (0.0576)
Employed	ref	ref	ref	ref
*Instrumental variable*				
Education				
No education	−0.864*** (0.0969)		− 0.514*** (0.0846)	
Primary	− 0.826*** (0.102)		− 0.429*** (0.0810)	
Secondary	−0.539*** (0.0777)		− 0.219** (0.0736)	
Higher	ref		ref	
*Summary Statistics*				
Number of observations	3853		9385	
Selected	2004		6171	
Non-selected	1849		3214	
Log likelihood	− 3007.572		− 8892.495	
Wald Chi- square	578.39		875.83	
Prob > Chi- square	< 0.0001		< 0.0001	
Athrho	1.659586		5,485,849	
Rho	9,301,614		.4994589	

Standard errors in parentheses

*p < 0.05

**p < 0.01

***p < 0.001

Women in Western, Volta, Eastern, Brong Ahafo, Northern, Upper East and the Upper West regions were significantly more likely to be covered by health insurance, compared to women in the Greater Accra region. Men in nine regions (Western, Central, Volta, Eastern, Ashanti, Brong Ahafo, Northern, Upper East and Upper West) were also significantly more likely to be covered for insurance, compared to men in the Greater Accra. Men and women who were not educated, or educated to primary and secondary levels were significantly less likely to be covered for health insurance, compared to those educated at tertiary level. On the effect of parity, multiparas women were significantly more likely to be covered for health insurance compared to women with parity levels of five and more. We found men and women religious status not to have any influence on their subscription for health insurance.

### Type of payment for health insurance and socio-demographic associations

Younger men and women aged 15–29 years were less likely to pay for NHIS premiums by themselves as presented in Table 3. However, increased age among women showed significant association with women who paid themselves to enroll under the NHIS. For instance, women aged 35–49 years were significantly associated with insured women who paid NHIS insurance premiums themselves, compared to women aged 30–34 years. Also, widowed women and women never in union were significantly associated with insured women who paid NHIS insurance premiums themselves, compared to married women (β = 0.789, *p* < 0.001 and β = 0.265, *p* < 0.01) respectively. Poorest, poorer and middle wealth groups for both men and women were less likely to pay NHIS premiums themselves to enroll for NHIS.

Regarding regional differentials and type of payment for insurance, men from two regions (Upper West and Upper East regions) were significantly more likely to have paid NHIS premiums by themselves, compared to men in the Greater Accra region (β = 1.033, *p* < 0.001 and β = 1.290, *p* < 0.001) respectively. In addition, men from Northern, Brong Ahafo, Ashanti, Eastern, Volta and Western regions were significantly associated with men who reported paying NHIS premiums by themselves, compared to men in Greater Accra region. Women from three regions (Upper West, Upper East and Volta) were significantly associated with women who reported paying NHIS premiums by themselves, compared to women in Greater Accra region (β = 0.418 *p* < 0.01, β = 0.336 *p* < 0.01 and β = 0.650 *p* < 0.001) respectively. Christian women and women who belonged to the Traditional/Spiritualist religion were significantly associated with women who paid NHIS insurance premiums themselves, compared to Islamic women (β = 0.160 *p* < 0.01 and β = 0.337 *p* < 0.05) respectively.

Parity status among women was found to be positively associated with the probability of self-payment for NHIS. Nulliparous women and multiparous women (1–2 and 3–4 births) were significantly associated with women who paid NHIS insurance premiums themselves, compared to women with 5+ births (β = 0.253 *p* < 0.01, β = 0.392 *p* < 0.001, and β = 0.147 *p* < 0.01) respectively. Overall, respondents who were unemployed and lived in rural areas were less likely to have paid NHIS insurance premiums themselves, compared to employed and urban residents respectively.

### Reasons for being uninsured and socio-demographic associations among men and women

Table 4 present socio-demographic associations among uninsured men and reasons why men were not covered for insurance. Men aged 15–19 years and those aged 35–39 years were significantly associated with uninsured men who stated that non-affordability of NHIS premiums as reason for not being insured, compared to men aged 30–34 years (β = 0.552 *p* < 0.001 and β = 0.243 *p* < 0.01) respectively.

**Table 4:** Probit Hackman model showing reasons for not being insured under the NHIS among men in Ghana

Variables	Male		Male		Male	
	Not covered by NHIS 1 = not covered, 0 = covered (selection)	Cannot afford NHIS (1 = yes, 0 = no)	Not covered by NHIS 1 = not covered, 0 = covered (selection)	Do not trust NHIS (1 = yes, 0 = no)	Not covered by NHIS 1 = not covered, 0 = covered (selection)	Do not need NHIS (1 = yes, 0 = no)
	β (SE)	β (SE)	β (SE)	β (SE)	β (SE)	β (SE)
Age						
15–19	− 0.361*** (0.105)	0.552*** (0.125)	−0.373*** (0.106)	−0.536** (0.206)	− 0.372*** (0.106)	−0.532** (0.180)
20–24	− 0.0500 (0.0981)	0.118 (0.115)	−0.0685 (0.0987)	−0.0991 (0.171)	− 0.0661 (0.0982)	−0.155 (0.138)
25–29	0.115 (0.0849)	−0.142 (0.102)	0.117 (0.0858)	0.0394 (0.152)	0.120 (0.0855)	−0.0418 (0.118)
30–34	ref	ref	ref	ref	ref	ref
35–39	−0.286** (0.0870)	0.243* (0.106)	−0.294*** (0.0881)	0.139 (0.181)	− 0.296*** (0.0881)	−0.0267 (0.142)
40–44	−0.197* (0.0920)	0.0418 (0.112)	− 0.203* (0.0935)	0.0459 (0.179)	− 0.208* (0.0936)	−0.0298 (0.137)
45–49	−0.388*** (0.0984)	0.194 (0.123)	−0.388*** (0.100)	− 0.347 (0.232)	− 0.390*** (0.101)	−0.00735 (0.164)
Marital status						
Never in union	0.192* (0.0971)	−0.141 (0.110)	0.176 (0.0983)	−0.109 (0.166)	0.171 (0.0980)	0.0423 (0.135)
Married	ref	ref	ref	ref	ref	ref
Widowed	0.253 (0.139)	0.138 (0.164)	0.266 (0.143)	−0.0772 (0.303)	0.276 (0.145)	−0.306 (0.210)
Wealth index						
Poorest	0.738*** (0.111)	0.336 (0.218)	0.738*** (0.113)	−0.0375 (0.311)	0.728*** (0.113)	−0.920*** (0.222)
Poorer	0.600*** (0.0994)	0.323 (0.182)	0.567*** (0.0990)	−0.202 (0.262)	0.558*** (0.0995)	−0.449* (0.195)
Middle	0.519*** (0.0882)	0.106 (0.148)	0.498*** (0.0877)	−0.283 (0.227)	0.492*** (0.0877)	−0.361* (0.167)
Richer	0.271*** (0.0770)	−0.00621 (0.113)	0.257*** (0.0770)	−0.129 (0.166)	0.256*** (0.0772)	0.00119 (0.137)
Richest	ref	ref	ref	ref	ref	Ref
Region						
Western	−0.341*** (0.0937)	0.193 (0.112)	− 0.340*** (0.0950)	−0.730*** (0.171)	−0.344*** (0.0943)	0.165 (0.131)
Central	−0.238* (0.0983)	0.412*** (0.110)	−0.231* (0.0996)	0.00423 (0.158)	−0.234* (0.0990)	−0.0858 (0.138)
Greater Accra	ref	ref	ref	ref	ref	ref
Volta	−1.002*** (0.106)	0.440** (0.147)	−0.982*** (0.108)	−0.903*** (0.259)	−0.980*** (0.108)	−0.371 (0.286)
Eastern	−0.624*** (0.0971)	0.408*** (0.123)	−0.623*** (0.0985)	−0.962*** (0.229)	−0.624*** (0.0980)	−0.124 (0.188)
Ashanti	−0.579*** (0.0933)	0.277* (0.125)	−0.573*** (0.0943)	0.0000631 (0.180)	−0.573*** (0.0944)	−0.0828 (0.171)
Brong Ahafo	−0.982*** (0.0983)	0.328* (0.159)	−0.973*** (0.0996)	−0.469 (0.243)	− 0.971*** (0.0995)	−0.0201 (0.243)
Northern	−1.158*** (0.111)	0.652*** (0.152)	−1.147*** (0.111)	−0.977*** (0.267)	−1.140*** (0.112)	0.0890 (0.273)
Upper east	−1.666*** (0.117)	1.311*** (0.162)	−1.668*** (0.118)	−1.245** (0.396)	−1.663*** (0.118)	−0.0650 (0.417)
Upper west	−1.623*** (0.119)	1.684*** (0.160)	−1.621*** (0.120)	−0.869* (0.392)	−1.616*** (0.120)	−0.711 (0.499)
Religion						
Christian	0.130* (0.0606)	0.0847 (0.0764)	0.114 (0.0616)	0.0184 (0.134)	0.120 (0.0617)	−0.0273 (0.0993)
Islam	ref	ref	ref	ref	ref	ref
Traditional/spiritualist	0.618*** (0.109)	−0.0445 (0.131)	0.587*** (0.108)	0.305 (0.271)	0.581*** (0.108)	−0.0757 (0.223)
No religion	0.539*** (0.103)	−0.198 (0.120)	0.543*** (0.106)	0.359 (0.204)	0.538*** (0.106)	0.0101 (0.170)
Residence						
Urban	ref	ref	ref	ref	ref	ref
Rural	−0.110 (0.0607)	−0.0749 (0.0747)	− 0.0966 (0.0603)	−0.171 (0.134)	− 0.0951 (0.0601)	0.189* (0.0959)
Parity						
Nulliparous	−0.0418 (0.121)	0.0211 (0.141)	−0.0490 (0.122)	0.188 (0.232)	−0.0482 (0.122)	−0.138 (0.179)
1–2	0.0371 (0.0875)	− 0.0799 (0.104)	0.0124 (0.0891)	0.201 (0.178)	0.00507 (0.0893)	−0.0833 (0.133)
3–4	−0.0486 (0.0789)	0.119 (0.0948)	−0.0689 (0.0799)	− 0.109 (0.169)	−0.0695 (0.0798)	− 0.101 (0.123)
5+	ref	ref	ref	ref	ref	ref
Employment						
Unemployed	−0.379*** (0.0689)	0.384*** (0.0863)	−0.391*** (0.0694)	−0.0159 (0.176)	− 0.390*** (0.0693)	−0.0786 (0.154)
Employed	Ref	Ref	Ref	Ref	Ref	Ref
*Instrumental variable*						
Education						
No education	0.837*** (0.106)		0.798*** (0.112)		0.809*** (0.110)	
Primary	0.802*** (0.107)		0.861*** (0.104)		0.873*** (0.101)	
Secondary	0.524*** (0.0864)		0.557*** (0.0872)		0.563*** (0.0847)	
Higher	ref		ref		ref	
*Summary Statistics*						
Number of observations	3854		3854		3854	
Selected	1849		1849		1849	
Non-selected	2005		2005		2005	
Log likelihood	− 3191.005		− 2752.658		− 3203.714	
Wald Chi- square	398.42		141.76		118.11	
Prob > Chi- square	< 0.0001		< 0.0001		< 0.0001	
Athrho	−1.572285		0.42878		−0.3853279	
Rho	−0.9173885		0.4043012		−0.3673255	

Standard errors in parentheses

*p < 0.05

**p < 0.01

***p < 0.001

Men in Upper West and Upper East regions were significantly more likely to have stated that non-affordability of NHIS premiums as reason for not being insured, compared to men in Greater Accra region (β = 1.684 *p* < 0.001 and β = 1.311 *p* < 0.001) respectively. Also, men in other three regions (Northern, Eastern and Central) were significantly associated with men who stated that non-affordability of NHIS premiums as reason for not being insured, compared to men in Greater Accra region. Unemployed men were significantly associated with men who stated that non-affordability of NHIS premiums as reason for not being insured, compared to employed men (β = 0.384 *p* < 0.001).

Young men aged 15–19 years were significantly less likely to state non-trust and no need for NHIS as reasons for not being insured compared to men aged 30–34 years. Also men in poorest, poorer and middle income groups were significantly less likely to state no need for NHIS as reason for not being insured, compared to men in the richest wealth group (β = − 0.920 *p* < 0.001, β = − 0.449 *p* < 0.01 and β = − 0.361 *p* < 0.01) respectively. Also, men in Western, Volta, Eastern, Northern, Upper East and Upper West regions were significantly less likely to state non-trust for the NHIS as reason for not being insured, compared to women in Greater Accra (see Table 4). Rural men were significantly associated with men who stated their no need for NHIS as reason for not insured, compared to urban men (β = 0.189 *p* < 0.01).

Table 5 presents the socio-demographic associations among uninsured women and reasons why some women are not covered for health insurance. Women aged 15–19 years were significantly associated with women who stated that non-affordability of NHIS premiums was reason for not being insured, compared to women aged 30–34 years. Women in poorest, poorer and middle income groups were significantly associated with those who stated non-affordability of NHIS premiums was reason for being uninsured, relative to women from richest income group (β = 0.729 *p* < 0.001, β = 0.609 *p* < 0.001 and β = 0.351 *p* < 0.01) respectively.

**Table 5:** Probit Hackman model showing reasons for not being insured under the NHIS among women in GhanaVariables

	Female		Female		Female	
	Not covered by NHIS 1 = not covered, 0 = covered (selection)	Cannot afford NHIS (1 = yes, 0 = no)	Not covered by NHIS 1 = not covered, 0 = covered (selection)	Do not trust NHIS (1 = yes, 0 = no)	Not covered by NHIS 1 = not covered, 0 = covered (selection)	Do not need NHIS (1 = yes, 0 = no)
	β (SE)	β (SE)	β (SE)	β (SE)	β (SE)	β (SE)
Age						
15–19	0.0978 (0.0696)	0.245* (0.110)	0.105 (0.0697)	−0.699** (0.239)	0.104 (0.0698)	− 0.527** (0.171)
20–24	0.203*** (0.0578)	−0.0519 (0.0911)	0.209*** (0.0578)	−0.109 (0.196)	0.209*** (0.0578)	−0.182 (0.146)
25–29	0.0299 (0.0523)	0.0334 (0.0815)	0.0303 (0.0523)	0.100 (0.160)	0.0304 (0.0523)	−0.232 (0.119)
30–34	ref	ref	ref	ref	ref	ref
35–39	−0.0496 (0.0537)	−0.0225 (0.0814)	− 0.0469 (0.0537)	0.134 (0.174)	− 0.0470 (0.0537)	−0.196 (0.124)
40–44	−0.00618 (0.0574)	− 0.170 (0.0896)	−0.00615 (0.0575)	− 0.00757 (0.197)	−0.00619 (0.0575)	0.106 (0.120)
45–49	−0.0663 (0.0613)	−0.0152 (0.0898)	− 0.0682 (0.0613)	0.0585 (0.205)	− 0.0684 (0.0613)	0.195 (0.131)
Marital status						
Never in union	0.228*** (0.0535)	−0.125 (0.0809)	0.228*** (0.0536)	0.237 (0.160)	0.227*** (0.0537)	0.149 (0.122)
Married	ref	ref	ref	ref	ref	ref
Widowed	0.183** (0.0605)	0.0205 (0.0873)	0.191** (0.0608)	−0.133 (0.214)	0.191** (0.0608)	0.0765 (0.136)
Wealth index						
Poorest	0.500*** (0.0714)	0.729*** (0.195)	0.503*** (0.0718)	−0.387 (0.389)	0.504*** (0.0718)	−0.446 (0.267)
Poorer	0.409*** (0.0623)	0.609*** (0.173)	0.420*** (0.0627)	−0.399 (0.310)	0.420*** (0.0628)	−0.195 (0.212)
Middle	0.341*** (0.0541)	0.351** (0.135)	0.345*** (0.0545)	−0.0857 (0.232)	0.345*** (0.0545)	−0.121 (0.176)
Richer	0.207*** (0.0491)	0.162 (0.104)	0.211*** (0.0492)	−0.203 (0.181)	0.211*** (0.0492)	−0.0628 (0.130)
Richest	ref	ref	ref	ref	ref	ref
Region						
Western	−0.383*** (0.0604)	0.0168 (0.1000)	− 0.382*** (0.0607)	0.462* (0.209)	−0.382*** (0.0606)	−0.125 (0.160)
Central	−0.0177 (0.0611)	0.0287 (0.0840)	−0.0216 (0.0613)	−0.132 (0.156)	− 0.0220 (0.0612)	0.124 (0.108)
Greater Accra	ref	ref	ref	ref	ref	ref
Volta	−0.665*** (0.0677)	0.138 (0.120)	− 0.666*** (0.0680)	−0.127 (0.307)	− 0.666*** (0.0679)	−0.266 (0.243)
Eastern	−0.506*** (0.0637)	0.264* (0.103)	−0.511*** (0.0639)	−0.349 (0.264)	− 0.512*** (0.0638)	−0.0284 (0.204)
Ashanti	0.0286 (0.0580)	−0.240** (0.0852)	0.0320 (0.0584)	− 0.555*** (0.169)	0.0317 (0.0582)	−0.252* (0.105)
Brong Ahafo	−0.843*** (0.0647)	0.0649 (0.142)	−0.843*** (0.0649)	−0.0934 (0.399)	− 0.843*** (0.0648)	−0.298 (0.294)
Northern	−0.821*** (0.0715)	0.720*** (0.104)	−0.812*** (0.0718)	−0.474 (0.396)	− 0.812*** (0.0718)	−0.565* (0.287)
Upper east	−0.822*** (0.0711)	−0.269 (0.166)	− 0.819*** (0.0712)	−0.155 (0.403)	− 0.819*** (0.0712)	−1.005** (0.343)
Upper west	−1.437*** (0.0804)	1.228*** (0.138)	−1.432*** (0.0807)	−0.347 (0.652)	−1.432*** (0.0806)	−0.806 (0.507)
Religion						
Christian	0.151*** (0.0416)	0.102 (0.0710)	0.153*** (0.0416)	−0.0722 (0.135)	0.153*** (0.0416)	0.0189 (0.109)
Islam	ref	ref	ref	ref	ref	ref
Traditional/spiritualist	0.351*** (0.0957)	0.468** (0.154)	0.358*** (0.0963)	−0.0601 (0.443)	0.358*** (0.0963)	0.0801 (0.276)
No religion	0.341*** (0.0855)	0.0928 (0.129)	0.354*** (0.0864)	0.162 (0.292)	0.353*** (0.0865)	0.170 (0.206)
Residence						
Urban	ref	ref	ref	ref	ref	ref
Rural	−0.0135 (0.0378)	−0.114* (0.0571)	−0.0137 (0.0378)	− 0.293* (0.123)	−0.0138 (0.0378)	0.121 (0.0871)
Parity						
No child	−0.103 (0.0710)	−0.0379 (0.111)	− 0.113 (0.0713)	−0.00485 (0.218)	− 0.113 (0.0713)	0.343* (0.172)
1–2	−0.211*** (0.0520)	−0.0834 (0.0920)	− 0.215*** (0.0522)	0.00494 (0.189)	−0.215*** (0.0522)	0.0830 (0.151)
3–4	−0.132** (0.0462)	0.0563 (0.0694)	− 0.134** (0.0464)	−0.116 (0.168)	− 0.134** (0.0464)	0.0586 (0.121)
5+	ref	ref	ref	ref	ref	ref
Employment status						
Unemployed	−0.0650 (0.0364)	0.152** (0.0530)	−0.0637 (0.0363)	0.203 (0.112)	−0.0637 (0.0364)	− 0.0972 (0.0871)
Employed	ref	ref	ref	ref	ref	ref
*Instrumental variable*						
Education						
No education	0.579*** (0.0745)		0.541*** (0.0821)		0.544*** (0.0821)	
Primary	0.485*** (0.0726)		0.451*** (0.0796)		0.454*** (0.0800)	
Secondary	0.275*** (0.0658)		0.246*** (0.0726)		0.249*** (0.0741)	
Higher	ref		ref		ref	
*Summary Statistics*						
Number of observations	9385		9385		9385	
Selected	3214		3214		3214	
Non-selected	6171		6171		6171	
Log likelihood	− 7034.182		− 5989.41		− 6610.028	
Wald Chi- square	407.8		102.26		125.46	
Prob > Chi- square	< 0.0001		< 0.0001		< 0.0001	
Athrho	−1.201377		0.0783509		0.0768847	
Rho	−0.8340742		0.078191		0.0767336	

Standard errors in parentheses

*p < 0.05

**p < 0.01

***p < 0.001

Women in Upper West were significantly more likely to have stated non-affordability of NHIS premiums was reason for being uninsured under the NHIS, compared to women from greater Accra region (β = 1.228 *p* < 0.001). In addition, women in Northern and Eastern regions were significantly associated with those who stated non-affordability of NHIS premiums was reason for being uninsured, relative to women in Greater Accra region. Unemployed women and women who belonged to the Traditionalist/Spiritualist religion were significantly associated with uninsured women who stated that non-affordability of NHIS premiums was reason for being uninsured, relative to employed and Islamic women respectively.

Women in Western region were significantly associated with women who stated non-trust of the NHIS as reason for being uninsured, relative to women in the Greater Accra region (β = 0.462 *p* < 0.05). Nulliparous women were significantly associated with women who stated they do not need NHIS as reason for being uninsured, relative to women with 5+ births (β = 0.343 *p* < 0.05).

## Discussion

As shown by our results, more than half of both men and women in our sample were covered by the NHIS. Single and widowed women were covered less under the NHIS, with the young, widowed, poorest and non-educated to be more likely to be uninsured because of non-affordability of NHIS premiums. Uninsured urban women reported non-trust as a reason for not being insured under the NHIS while nulliparous women were more likely to be uninsured because of their perceptions that they do not need NHIS. Inequity concerns for social health insurance have already been reported in Ghana, as well as in other Sub-Saharan African countries, e.g. South Africa and Tanzania [21]. Further discussions of the findings based on the study aim are presented in the next sub-sections.

### Health insurance coverage

Coverage for health insurance was lower among women never in union or widowed relative to married women. Singlehood and widowed may thus present a level of vulnerability and inability to enroll to the NHIS. In low-middle-income countries (LMIC), widowhood represents a ‘vulnerable’ group [22] and may have influenced women’s low coverage for health insurance as found from this study in Ghana. Married women may have enhanced ability to pay for insurance to avoid wider catastrophic household spending later as corroborated by other studies [23, 24]. In addressing equity concerns regarding access and coverage for NHIS, widowed and single women in low socio-economic income groups are likely to have decreased ability to enroll to the NHIS.

Under Ghana’s NHIS exemption policy, widows may be exempted, or covered for health insurance through the Livelihood Empowerment Against Poverty Program (LEAP), a pro-poor social intervention policy [25]. The LEAP program can be reformed to ensure better targeting and inclusion of vulnerable groups. Proxy means testing (PMT), participatory welfare ranking (PWR), and geographic targeting (GT) for pro-poor groups can assist to improve efficiency and equity needs under the NHIS in Ghana [26]. We found that as the proportion of non-educated men and women increases, more men and women remain uninsured under the NHIS. Previous studies [11, 16, 18, 27] corroborate our findings by establishing that lower educated men and women are more likely to be uninsured. Literacy improvements are associated with better health outcomes [28] and the potential to reduce income-related inequalities [29]. A mix of education and health policies by the NHIS is needed to create more awareness of the benefits for NHIS enrolments. Literacy and advocacy interventions can address current effects of education on NHIS enrolments.

We posit that increased parity (5+ births) particularly among lower socio-economic groups have the potential to reduce a woman’s ability to pay for health insurance. This is possible since a woman is likely to spend extra money at home to meet other caring needs of her family and children, instead of paying to enroll for NHIS. Given that NHIS enrolment among women can improve focused maternity care in Ghana [30], and can facilitate interventions to address the needs among women with high parities. Unemployed women and men were more likely to be covered for insurance, which could have been because of a positive re-enforced health seeking attitude to meet current as well as avoid any potential catastrophic health expenditures in the future.

Overall, while this study found that the above factors determine coverage for health insurance, other environmental factors not measured in this study have also been found to play a significant role in influencing an individual ability and willingness to take up health insurance. In Africa, studies [31, 32] have found that broad environmental factors, such as health services availability and the quality of health services, provided to influence health insurance uptake. Other studies [33, 34] in settings outside Africa have also established the broader goal of working to improve the social determinants of health as plausible policies for meeting UHC goals [35].

### Type of payment among men and women insured under the NHIS in Ghana

We found that older women were more likely to pay themselves for health insurance, as corroborated in other studies [36–38]. Older persons may have better economic opportunity and social networks to be able to pay to be enrolled [39, 40]. The willingness to obtain insurance to meet chronic conditions health needs is often associated with older age [36, 41]. Currently, older persons (70+ years) in Ghana pay for card processing and renewal under the NHIS. Premium payments are the only exemptions for older persons. Despite premium fee exemptions for older persons in Ghana, NHIS enrolments may remain limited for older persons due to socio-economic factors such as low education, and facility type among others [42]. The minimum benefit package for older persons excludes rehabilitative services and surgeries related to heart and brain, and treatments for chronic kidney conditions [4]. An insurance model that allows co-payments to meet a broad range of health care services outside the minimum benefit package is essential to meet the Universal Health Coverage (UHC) demands and address health care utilization challenges under the NHIS.

Although women never in union and those widowed were less often covered for insurance as reported earlier, those that were covered under the NHIS were more likely to have paid themselves to be enrolled. This may indicate the absence of spousal and other family support to meet NHIS enrolment needs for single and widowed women. In the absence of social support for disadvantaged individuals from these two groups (single or widowed women), enrolment disparities are likely to arise. As expected, men and women who were insured but belonged to the poorest, poorer and middle-wealth groups were less likely to have paid themselves to enroll to the NHIS, given the predictive influence of wealth on insurance enrolments in other studies in Ghana [17, 43, 44].

We also observed increased chances for nulliparous and multipara women (1–4 births) to have paid themselves to be enrolled under the NHIS, relative to women with > 5 parities. Nulliparous women and women with 1–4 births may have enhanced economic capacity compared to women with higher parities (> 5) as earlier explained. The burden of enrolling for NHIS may be greater for women with higher parities, and who reside in economically vulnerable households in Ghana as found in this study. In settings where a woman may lack self-autonomy regarding decisions on health insurance and general health care seeking [45], increased parity levels have the tendency to discourage women taking up health insurance given other household competing demands for other dimensions of care. Large household size may present high inter-individual inequality outcomes [46], affecting income allocations for health insurance by different members at the household level. This finding is further corroborated by a study in Ethiopia that reported that household members respond to conditions of ill-health differently when the need to seek for care arises, given that household resource allocations may be unequal [47].

### Reasons for not being insured under NHIS

Non-affordability of NHIS premiums was a reason for young and unemployed men and women to remain uninsured under the NHIS. Unemployed persons may have limited capacity to be insured because opportunities for employment and health are intrinsically related [48], and thus may influence an individual ability and willingness to be insured. Higher educational status creates opportunity for employment and further translates to better health insurance coverage at individual and population level [49–51]. Health policy reforms for meeting the UHC goals in Ghana will require measures that address existing horizontal equity concerns regarding direct and indirect effects of employment and educational attainments on NHIS enrolments in Ghana. Geographically, non-affordability for NHIS premiums as a reason for being uninsured were found among men in the Upper West and Upper East regions and among women in the Upper West region. Uninsured men and women from two other regions: Northern and Eastern regions were uninsured under the NHIS because of their inability to afford NHIS premiums. Uninsured men from Central region were also more likely to report inability to afford NHIS premiums as a reason for not being insured under the NHIS. Among women who were uninsured from the Western region, perceptions of non-trust in the NHIS were major reason for being uninsured.

Our findings on geographic differentials, non-affordability to pay NHIS premiums to enroll under the NHIS in Ghana is consistent with evidence that shows more lower socio-economic income groups reside in these areas in Ghana [52]. To reduce geographic and economic barriers for enrolling under the NHIS in Ghana, there is a need for pro-poor social support systems to enable residents in these areas of Ghana to enroll for insurance. Although the data did not provide reasons for non-trust under the NHIS as reported among women from Western region, poor accountability and transparency issues under the NHIS are documented in other studies [9, 10] and may lead to trust concerns.

At the health system level, reducing long waiting days and time to obtain a NHIS membership cards, could encourage to take up health insurance. Further decentralizing the operations of NHIS that allow for real-time registration and renewals of NHIS membership can be a positive precursor to improve trust and influence client’s ability to overcome physical and economic barriers for enrolling health insurance. Furthermore, rural uninsured men perceptions of no need for health insurance may be informative of the general non-trust and public confidence for the NHIS reported in previous studies in Ghana [20, 53]. Nulliparous uninsured women in this study often reported that they do not need NHIS as a reason for being uninsured. This could be because nulliparous women may perceive low susceptibility for seeking health care, which may influence their perceptions of no need for insurance.

This study has limitations. The use of observations data made it impossible to draw causal inferences and to include all policy, economic and environmental factors that influence coverage for health insurance. We limited ourselves to the use of non-trust, non-affordability of premiums and no need for insurance to assess the reasons of being uninsured. We could not ascertain from the dataset if the payment response “pay myself” meant that the individual use own funds. Hence, the purchasing parity based on type of payment should be interpreted with caution. Because the 2014 GDHS covered men aged 15–59, our sample was limited to men and women aged 15–49 to ensure comparability of the data. In addition, we were not able to statistically test the difference between the gender groups because the data were collected separately for men and women. The samples were also drawn independently of each other. Hence, it was not possible to pool the two datasets together.

## Conclusion

Poor health insurance coverage among socially vulnerable groups, such as single and widowed women, poorest and non-educated persons that we found in this study is indicative of the inequity in the NHIS. Targeted schemes and social interventions are important to ensure universal access to health care under the NHIS. Such schemes and interventions could provide social support for membership enrolment for vulnerable identified groups in both urban and rural areas. Additionally, we found that non-trust in the NHIS is a reason for some women to remain uninsured. This indicates the need of tailored policies targeted at reducing non-transparency, delays in processing membership enrolments and encouraging enrolments to the NHIS. Further, decentralizing the operations of NHIS could allow for real-time registration, renewals of NHIS membership and feedback on service quality by service providers. This can be a positive precursor to improve trust and influence client’s ability to overcome physical and economic barriers for enrolling to a health insurance scheme. A mix of education and health program interventions targeted at creating more awareness of the benefits for NHIS enrolments, through literacy and advocacy interventions, can support the enrolments, build trust and address current enrolment challenges driven by socio-demographic factors in Ghana.

## Data Availability

Demographic Health Data is available online from the DHS MEASURE Program upon request. Authors are willing to share the data file used in this study upon request.

## Abbreviations

GDHS: Ghana Demographic Health Survey
GT: Geographic targeting
NHIS: National Health Insurance Scheme
OOP: Out-of-pocket payments
PMT: Proxy means testing
PWR: Participatory welfare ranking
UHC: Universal Health Coverage
